# Disturbance in cerebral blood microcirculation and hypoxic-ischemic microenvironment are associated with the development of brain metastasis

**DOI:** 10.1093/neuonc/noae094

**Published:** 2024-06-04

**Authors:** Jenny Roesler, Daniel Spitzer, Xiaoxiong Jia, Synnøve Nymark Aasen, Kathleen Sommer, Bastian Roller, Niels Olshausen, Nils R Hebach, Nawid Albinger, Evelyn Ullrich, Ling Zhu, Fan Wang, Jadranka Macas, Marie-Therese Forster, Joachim P Steinbach, Lisa Sevenich, Kavi Devraj, Frits Thorsen, Matthia A Karreman, Karl H Plate, Yvonne Reiss, Patrick N Harter

**Affiliations:** Goethe University, University Hospital, Institute of Neurology (Edinger Institute), Frankfurt, Germany; Goethe University, University Hospital, Institute of Neurology (Edinger Institute), Frankfurt, Germany; Tianjin Neurosurgical Institute, Tianjin Huanhu Hospital, Tianjin, China; Tianjin Key Laboratory of Cerebral Vascular and Neurodegenerative Diseases, Tianjin Huanhu Hospital, Tianjin, China; Goethe University, University Hospital, Institute of Neurology (Edinger Institute), Frankfurt, Germany; Neurosurgery Department, Tianjin Huanhu Hospital, Tianjin, China; Department of Oncology and Medical Physics, Haukeland University Hospital, Bergen, Norway; Department of Biomedicine, Kristian Gerhard Jebsen Brain Tumour Research Centre, University of Bergen, Bergen, Norway; Goethe University, University Hospital, Institute of Neurology (Edinger Institute), Frankfurt, Germany; Goethe University, University Hospital, Dr. Senckenberg Institute for Neurooncology, Frankfurt, Germany; Goethe University, University Hospital, Institute of Neurology (Edinger Institute), Frankfurt, Germany; Clinical Cooperation Unit Neurooncology, German Cancer Consortium (DKTK), German Cancer Research Center (DKFZ), Heidelberg, Germany; Clinical Cooperation Unit Neurooncology, German Cancer Consortium (DKTK), German Cancer Research Center (DKFZ), Heidelberg, Germany; Frankfurt Cancer Institute (FCI), Frankfurt, Germany; Department of Pediatrics, Experimental Immunology and Cell Therapy, Goethe University, University Hospital, Frankfurt, Germany; Frankfurt Cancer Institute (FCI), Frankfurt, Germany; Department of Pediatrics, Experimental Immunology and Cell Therapy, Goethe University, University Hospital, Frankfurt, Germany; Goethe University, University Hospital, Institute of Neurology (Edinger Institute), Frankfurt, Germany; Goethe University, University Hospital, Institute of Neurology (Edinger Institute), Frankfurt, Germany; Goethe University, University Hospital, Institute of Neurology (Edinger Institute), Frankfurt, Germany; Department of Neurosurgery, Goethe University, University Hospital, Frankfurt, Germany; German Cancer Research Centre (DKFZ), Heidelberg, Germany; German Cancer Consortium (DKTK) Partner Site Frankfurt/Mainz, Frankfurt, Germany; Frankfurt Cancer Institute (FCI), Frankfurt, Germany; Goethe University, University Hospital, Dr. Senckenberg Institute for Neurooncology, Frankfurt, Germany; Institute for Tumor Biology and Experimental Therapy, Georg-Speyer-Haus, Frankfurt am Main, Frankfurt, Germany; German Cancer Research Centre (DKFZ), Heidelberg, Germany; German Cancer Consortium (DKTK) Partner Site Frankfurt/Mainz, Frankfurt, Germany; Frankfurt Cancer Institute (FCI), Frankfurt, Germany; Goethe University, University Hospital, Institute of Neurology (Edinger Institute), Frankfurt, Germany; Department of Biological Sciences, Birla Institute of Technology and Science, Pilani, Hyderabad, India; Department of Biomedicine, Molecular Imaging Center, University of Bergen, Bergen, Norway; Department of Neurosurgery, Qilu Hospital of Shandong University and Brain Science Research Institute, Shandong University, Jinan, China; Department of Neurosurgery, Haukeland University Hospital, Bergen, Norway; Neurology Clinic and National Center for Tumor Diseases, University Hospital Heidelberg, Heidelberg, Germany; Clinical Cooperation Unit Neurooncology, German Cancer Consortium (DKTK), German Cancer Research Center (DKFZ), Heidelberg, Germany; German Cancer Research Centre (DKFZ), Heidelberg, Germany; German Cancer Consortium (DKTK) Partner Site Frankfurt/Mainz, Frankfurt, Germany; Frankfurt Cancer Institute (FCI), Frankfurt, Germany; Goethe University, University Hospital, Institute of Neurology (Edinger Institute), Frankfurt, Germany; German Cancer Research Centre (DKFZ), Heidelberg, Germany; German Cancer Consortium (DKTK) Partner Site Frankfurt/Mainz, Frankfurt, Germany; Frankfurt Cancer Institute (FCI), Frankfurt, Germany; Goethe University, University Hospital, Institute of Neurology (Edinger Institute), Frankfurt, Germany; Center for Neuropathology and Prion Research, Faculty of Medicine, Ludwig-Maximilians-Universität München, Munich, Germany; German Cancer Research Centre (DKFZ), Heidelberg, Germany; German Cancer Consortium (DKTK) Partner Site Frankfurt/Mainz, Frankfurt, Germany; Frankfurt Cancer Institute (FCI), Frankfurt, Germany; Goethe University, University Hospital, Institute of Neurology (Edinger Institute), Frankfurt, Germany

**Keywords:** angiopoietin-2, anti-angiogenic therapy, brain metastases, perivascular niche, VEGF

## Abstract

**Background:**

Brain metastases (BM) constitute an increasing challenge in oncology due to their impact on neurological function, limited treatment options, and poor prognosis. BM occurs through extravasation of circulating tumor cells across the blood-brain barrier. However, the extravasation processes are still poorly understood. We here propose a brain colonization process which mimics infarction-like microenvironmental reactions, that are dependent on Angiopoietin-2 (Ang-2) and vascular endothelial growth factor (VEGF).

**Methods:**

In this study, intracardiac BM models were used, and cerebral blood microcirculation was monitored by 2-photon microscopy through a cranial window. BM formation was observed using cranial magnetic resonance, bioluminescent imaging, and postmortem autopsy. Ang-2/VEGF targeting strategies and Ang-2 gain-of-function (GOF) mice were employed to interfere with BM formation. In addition, vascular and stromal factors as well as clinical outcomes were analyzed in BM patients.

**Results:**

Blood vessel occlusions by cancer cells were detected, accompanied by significant disturbances of cerebral blood microcirculation, and focal stroke-like histological signs. Cerebral endothelial cells showed an elevated Ang-2 expression both in mouse and human BM. Ang-2 GOF resulted in an increased BM burden. Combined anti-Ang-2/anti-VEGF therapy led to a decrease in brain metastasis size and number. Ang-2 expression in tumor vessels of established human BM negatively correlated with survival.

**Conclusions:**

Our observations revealed a relationship between disturbance of cerebral blood microcirculation and brain metastasis formation. This suggests that vessel occlusion by tumor cells facilitates brain metastatic extravasation and seeding, while combined inhibition of microenvironmental effects of Ang-2 and VEGF prevents the outgrowth of macrometastases.

**Key Points**

- Brain metastasis extravasation is facilitated by alterations in cerebral blood microcirculation, Ang-2, and VEGF.
- Combined inhibition of microenvironmental effects of Ang-2 and VEGF prevents the outgrowth of macrometastases in the brain.

**Importance of the Study**

Despite recent advances in the understanding of brain colonization processes of cancer cells in the brain, the complex interplay between cancer and microenvironmental cells is poorly understood. Here, we demonstrate an Ang-2-dependent relationship of focal stroke-like cellular mechanisms which might facilitate cancer cell extravasation into the brain. This opens a new perspective on the biological processes of cancer cell extravasation in the brain, but also on treatment options, as the outgrowth of macrometastases was successfully inhibited by combined treatment targeting Ang-2 and VEGF.

Brain metastases (BM) develop in approximately 20% of all patients suffering from cancer, with the highest incidence in those with lung carcinoma, melanoma, renal, breast, and colorectal carcinoma.^1,2^ Despite continuous advancements in cancer treatment, brain metastasis substantially contributes to poor patient prognosis as it is still associated with high morbidity and mortality.^3^ Crucial steps in the early brain metastatic cascade include the transmigration of circulating tumor cells across the blood-brain barrier (BBB), seeding, and colonization in the perivascular pre-metastatic niche.^4–7^ The perivascular niche may provide oxygen, nutrients, and attachment to invading cancer cells, further promoting the initiation and progression of BM.^8,9^ Real-time imaging has demonstrated that cancer cells invading the brain parenchyma seed in the perivascular niche in close contact with capillary endothelial cells and adhere to the abluminal side of microvessels after extravasation.^6^ Lung and breast carcinoma, as well as melanoma cells, have been shown to exploit preexisting brain microvessels as main tracks for cancer cell invasion and metastasis outgrowth.^6,7^

Transmigration of cancer cells through endothelial barriers is a crucial event in BM formation. Therefore, angiogenic factors such as Angiopoietin-2 (Ang-2) and vascular endothelial growth factor (VEGF) have previously been studied. Both mediators are involved in the angiogenic switch at the early time points of the brain metastatic cascade^10,11^ and may contribute to tumor cell proliferation and metastasis formation in the perivascular pre-metastatic niche.^5,12,13^ Indeed, upregulation of both, Ang-2 in capillary endothelial cells and VEGF in tumor cells, can activate the brain microvasculature, leading to BBB impairment that further promotes the transmigration of cancer cells through the vascular wall to seed and colonize in the perivascular pre-metastatic niche in the brain.^5,13^ These microenvironmental processes are likely to occur in the context of hypoxia, which may trigger overexpression of Ang-2 and VEGF. This further contributes to the modulation of the local perivascular microenvironment generating a supporting milieu, designated as the pre-metastatic niche.^14–17^ Moreover, there is evidence that increased expression of Ang-2 in endothelial cells of co-opted blood vessels facilitates vascular destabilization and regression, leading to hypoxia-induced upregulation of Ang-2, VEGF, and subsequent induction of angiogenesis at the tumor margin.^12^ Several preclinical studies have demonstrated that therapeutic targeting of Ang-2 and/or VEGF can slow tumor growth and reduce tumor angiogenesis. This includes inhibition of the Ang-2-Tie-2 and/or VEGF signaling pathway by antibodies and peptide-Fc fusion proteins,^15–21^ or bispecific antibody-associated blockage of Ang-2 and VEGF.^22^

Brain tumor angiogenesis is well studied, however, early steps in the brain metastatic cascade are only partly understood. Kienast and coworkers described the importance of intraluminal cancer cell arrest as a crucial step for brain colonization.^6^ Later, Follain and coworkers studied hemodynamic forces as regulators of circulating cancer cell arrest, adhesion, and cancer cell extravasation.^23^ Furthermore, local blood coagulation has been described as a driver of cancer cell arrest and brain metastasis formation.^23,24^ Recently, Karreman and coworkers described an active remodeling process of the capillary endothelium as a promoter of cerebral metastatic colonization.^25^

In this study, we investigated early microenvironmental changes in the pre-metastatic niche in the brain. We monitored cerebral blood microcirculation through a cranial window using intravital 2-photon microscopy and investigated vascular permeability factors including Ang-2 in brain microvessels in vivo and measured the impact on transendothelial electrical resistance (TEER) in vitro. Moreover, we assessed VEGF expression in tumor cells in vitro, and further investigated whether these processes are more likely to occur in a hypoxic environment. In an Ang-2 gain-of-function (GOF) BM model we show an increased BM burden. Assuming that an increased Ang-2 and VEGF expression may change the local perivascular microenvironment towards a supporting pre-metastatic environment at the early stages of the metastatic cascade, we further tested whether combined treatment with AMG 386^26^ (a peptibody binding to angiopoietin-1 and angiopoietin-2) and aflibercept^27,28^ (a fusion protein containing domains of VEGFR1 and VEGFR2, thereby acting as “VEGF Trap”) at early stages may inhibit melanoma and breast carcinoma BM formation.

## Materials and Methods

### Tissue Microarrays

Formalin-fixed, paraffin-embedded (FFPE) tissue blocks from 191 brain metastasis patients were retrieved from the UCT tumor bank, Goethe-University, Frankfurt am Main, Germany. All studies on human brain tumor tissue were approved by the Institutional Review Board of the Ethical Committee at the University Hospital Frankfurt (project number: G 04/09, SNO-02-2017). For extended clinical data please see Supplementary Table 1.

### Animal care and handling

Six- to eight-week-old female athymic nude mice (Crl:NU(NCr)-Foxn1nu) were used and purchased from Charles River Laboratories (USA). Animals were housed under standard specific-pathogen-free conditions in a temperature-, humidity- and light-cycle-controlled facility (22 ± 2°C; 50% ± 10%; 12 hours light/dark cycle) with free access to food and water. The Ang-2 GOF mouse line comprising a Tie1 tTA driver and a TetOS human Ang-2 responder transgene were generated as described previously.^20,29^ For intravital 2-photon microscopy, NOD-SCID gamma (NSG, at least 10 weeks old) mice were used. All experiments were strictly conducted in accordance with the German Protection of Animals Act and in compliance with the recommendations in the Guide for Care and Use of Laboratory Animals of the National Institutes of Health (approval numbers FK/ 1085; 35-9185.81/G-220/16 and 35-9185.81/G-273/19; supplementary methods).

### Generation of Experimental Brain Metastasis and Treatment Regime

All in vivo experiments were performed using H1_DL2 or JIMT-1 brain-homing cells. Six- to eight-week-old female athymic FoxN1 nude mice (Crl:NU(NCr)-Foxn1^*nu*^) were used for the experiments. To generate BM, 1 × 10^5^ H1_DL2 or JIMT-1 brain-homing tumor cells were injected into the left cardiac ventricle, as previously described^30,31^ (Supplementary Methods). For immunohistochemistry, formalin-fixed and paraffin-embedded tissue collected from our earlier experimental studies was used.^32,33^ Three anti-angiogenic treatment regimens were commenced 24 hours before intracardiac (i.c.) tumor cell injection, including subcutaneous (s.c.) administration of AMG 386 (5.6 µg/g body weight) or aflibercept (25 µg/g body weight) alone,^19,27^ or AMG 386 + aflibercept (A + A, 5.6 µg/g body weight + 25 µg/g body weight). Subcutaneous administration of anti-angiogenic agents at the indicated dosages was repeated on day 5 after i.c. tumor cell injection, and subsequently twice a week until the endpoint was reached. Animals in the respective control groups received s.c. injections of PBS at the indicated time points as stated above. Syngeneic Ang-2 GOF experiments were performed by intracardiac injection of 50 000 99LN murine breast cancer cells.^34^

### MR Imaging, Bioluminescent Imaging, and BM quantification

BM progression of JIMT-1 and H1_DL2 cells was monitored by MRI measurements (JIMT-1: JR, XJ, LS 2 and 4 weeks after i.c. injection of tumor cells; H1_DL2: SNA, FAT 4 weeks after i.c. injection of tumor cells). MR imaging was performed using a 7 Tesla Small Animal MR Scanner (PharmaScan, Bruker, Ettlingen, Germany) equipped with a 72 mm quadrature transmit coil and a 4 channels mouse brain array receive coil. The development of 99LN brain metastasis was monitored by MRI at time points 28 days post injection (p.i.), 35 days p.i. and 42 days p.i.. For more information on MR Imaging parameters please see Supplementary Methods.

Images were exported as Dicom files and analyzed using ITK-SNAP software (Version 3.6.0).^31^ All metastases recognized at the 11–12 images for each brain were marked and differentially labeled. Metastases volume in mm^3^ was calculated automatically by the software.

For bioluminescent imaging, tumor-bearing mice were injected intraperitoneally with luciferin (1.5 mg/mL) 2 weeks post intracardiac injection. Mice were anesthetized and imaged using a IVIS Lumina II charge-coupled device imaging system (Caliper, Perkin-Elmer). Regions of interest were defined as “whole body,” “brain,” “spinal” and “femur.” Statistical analysis was performed using a Student’s *t*-test.

### Blood Velocity Measurement and 2-Photon Microscopy

Inhouse-bred, female NOD-scid IL2rgnull mice (NSG) older than 8 weeks were anesthetized with ketamine/xylazine, and cranial window implantation was performed as described previously.^6^ Minimum of 3 weeks following surgery, 500 000 JIMT-1 Br tumor cells, suspended in phosphate-buffered saline (PBS), were directly injected into the left ventricle of the NSG mice.

Multiphoton Laser Scanning Microscopy (MPLSM) imaging was performed 1 day after heart injection with tumor cells. Mice were scanned for cancer cells arrested in the vasculature on day 1 or day 1 through 12, respectively. For details please see Supplementary Methods.

### Tissue Preparation and Immunostaining

FFPE human tissue microarrays (TMAs) and metastatic mouse brain samples were cut into 3 µm thin sections on a microtome (Leica Microsystems, Nussloch GmbH, Nussloch, Germany) and further processed for hematoxylin and eosin (H&E) and IHC stainings. Details of used primary antibodies and staining procedure are depicted in Supplementary Methods section.

### Cell Viability and Toxicity Assays

For assessment of potential anti-proliferative and toxic effects of AMG 386 and aflibercept on human brain metastatic tumor cells in vitro, we conducted the crystal violet assay and 3- (4,5-Dimethylthiazol-2-yl)-2,5-diphenyltetrazolium bromide (MTT) reduction assay. Details of H1_DL2 and JIMT-1 experiments are depicted in Supplementary Methods section.

### Real-Time Quantitative PCR, Hypoxia Experiments.

Data analysis of microvessel isolation of stroke experiments were conducted as described by Spitzer et al.^35,36^ Total RNA was extracted according to the protocol of the RNeasy Mini Kit (Qiagen, Hilden, Germany) from 3, 6, and 12 hours after induction of hypoxia. Hypoxia was induced with Gas Pak pouches (Becton- Dickinson, Heidelberg, Germany). The real-time quantitative PCR was performed with primers against VEGF and the housekeeping gene Ribosomal protein lateral stalk subunit P0 (Rplp0; Supplementary Methods).

### Transendothelial Electrical Resistance

TEER measurements were performed as previously described.^29^ Details are depicted in the Supplementary Methods section.

### Statistical Analysis

Data are represented as histogram bars or dot plots with underlying bar graphs showing mean ± SEM (standard error of the mean). The statistical details for each experiment can be found in the figure legend (including sample size, statistical test, and *P*-values). A *P*-value < .05 was considered statistically significant. Quantitative and statistical analyses were performed using GraphPad Prism 9 software (GraphPad Software, Inc), JMP software (SAS) as well as R Statistical Software (v4.2.3).

## Results

### Cancer Cell Vessel Occlusion Leads to Alterations of Cerebral Blood Microcirculation and Focal Infarction-Like Microenvironmental Reactions in the Pre-metastatic Niche

**Figure 1.**
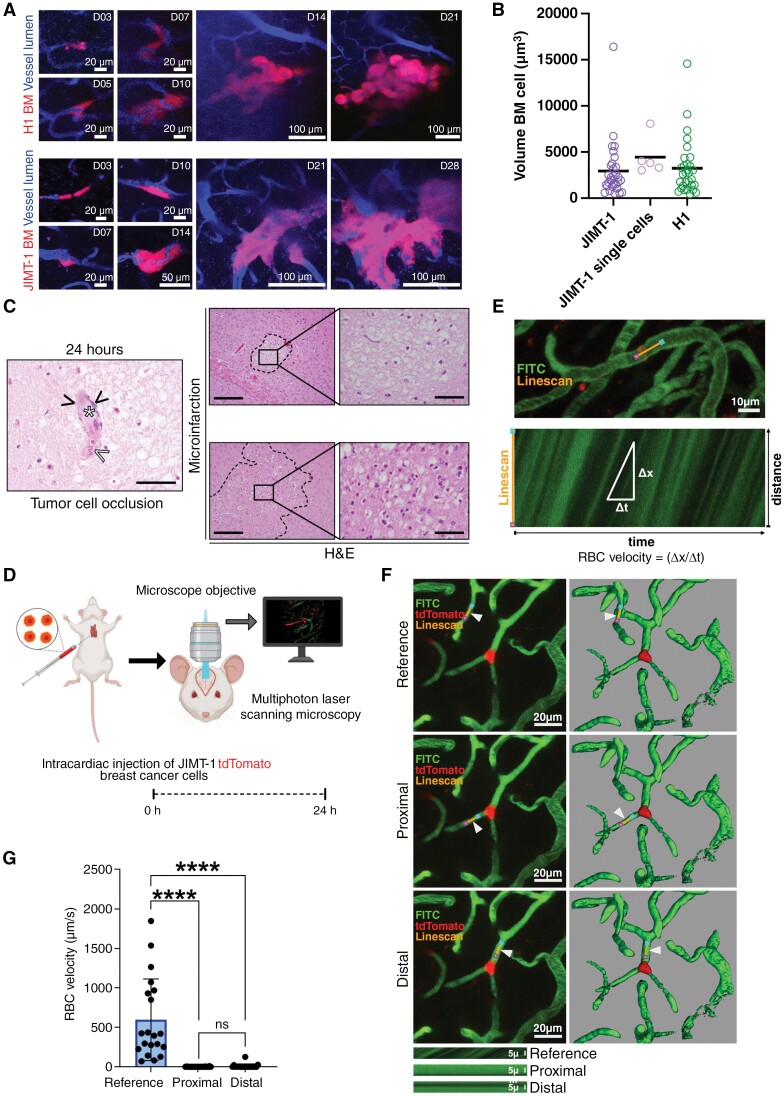
(A) Brain homing pattern of H1 melanoma and JIMT- breast cancer brain metastases between days 3 and 21 or 28, respectively. (B) Cancer cell thrombi volume was measured using reference volume data from electron microscopy-proven JIMT-1 single cells. (C) H&E staining showing microinfarction and a cancer cell (white asterisk) occluding a brain vessel (black arrowheads, scale bar 50 µm) close to a microinfarction with hypoxic eosinophilic neurons. White arrowhead depicting blood clotting. 24-hour time point showing examples of microinfarctions (images right, overview scale bar 200 µm, insert scale bar 50 µm). (D) Schematic summary illustrating blood flow velocity measurement experiments, depicting intracardiac injection of tdTomato expressing JIMT-1 breast cancer cells followed by in vivo multiphoton laser scanning microscopy imaging 24 hours post-intracardiac injection. (E) Example of a linescan trajectory (orange line), with pink and blue points marking linescan start and stop positions, respectively. Iterative scanning produces a time–space plot (E, bottom) where the angle of the streaks formed by moving red blood cells (RBC) is related to the speed of flow. The vertical dimension is distance along the linescan path, and the horizontal dimension is time. (F) Representative z-projections (F, left) and reconstructed 3D (F, right) in vivo images of FITC-dextran (fluorescein isothiocyanate-Dextran) positive blood vessels demonstrating tdTomato expressing JIMT-1 breast cancer cell arrested in microvessels, and line scan trajectories positioned in reference microvessel and in microvessel proximal and distal of the arrested tumor cell, indicated by arrowheads; examples of raw linescan data (kymographs; F, bottom) from microvessels collected parallel to the blood flow proximal and distal of the arrested tumor cell, and in a neighboring, independent microvessel (reference) of similar size and shape. (G) RBC velocity (µm/s) in neighboring, independent microvessels (reference), and in microvessels proximal and distal of arrested tumor cell in mice 24 hours post-intracardiac injection, *n* = 20; ****P < .0001 and not significant (ns) *P* > .05. *Indicates 2-tailed, unpaired *t*-test with Welch’s correction when variances were significantly different based on F-test.

We hypothesized that microvascular cancer cell occlusion and therefore focal alterations of cerebral blood circulation lead to hypoxic and ischemic events. We monitored cancer cell extravasation into the brain using MPLSM of H1 and JIMT-1 cancer cells. Both cell lines showed intravascular cell arrest in brain microvessels before extravasation and micrometastases formation (Figure 1A). Arrested cancer cell thrombi are formed by 1 or 2 cancer cells, based on the volume of arrested intravascular cancer cells measured by MPLSM and comparing this to the volume of single cells identified by correlative MPLSM and electron microscopy (Figure 1B). We investigated tissue specimens from intracardially injected H1_DL2 or PBS in immune-compromised mice followed by histopathological analysis 24 hours, 1 and 2 weeks post intracardiac injection (Figure 1C)^32,33^ (scheme of this experimental setup is illustrated in Supplementary Figure 1A). Importantly, histopathological analysis 24 hours post-tumor cell injection revealed focal microinfarctions in the brain parenchyma with obvious eosinophilic neurons as signs of early hypoxic damage (Figure 1C). Furthermore, we observed cancer cells occluding blood vessels accompanied by clot formation close to the microinfarctions (Figure 1C). A map of 24-hour microinfarctions and overlay with later tumor colonization (Supplementary Figure 1B) revealed a partial overlay of ischemic regions and later metastatic foci, suggesting that early colonization of brain metastatic cells might be associated with an early hypoxic microenvironment. Cancer cell vessel occlusions might therefore be associated with severe alterations of red blood cell microcirculation in the brain. We therefore performed 2-photon microscopy through a cranial window which enabled us to measure blood cell velocity during early brain colonization (Figure 1D-G).^25^ We saw that JIMT-1 tumor cells, which expressed a fluorescent protein occluded brain microvessels, leading to a significant reduction of red blood cell flow in the brain (Figure 1F, G and Supplementary Video 1), explaining focal hypoxia and ischemia.

### Early Brain Colonization Focally Mimics Infarction-Like Lesions With Upregulation of hypoxia-Induced Ang-2 and VEGF

**Figure 2.**
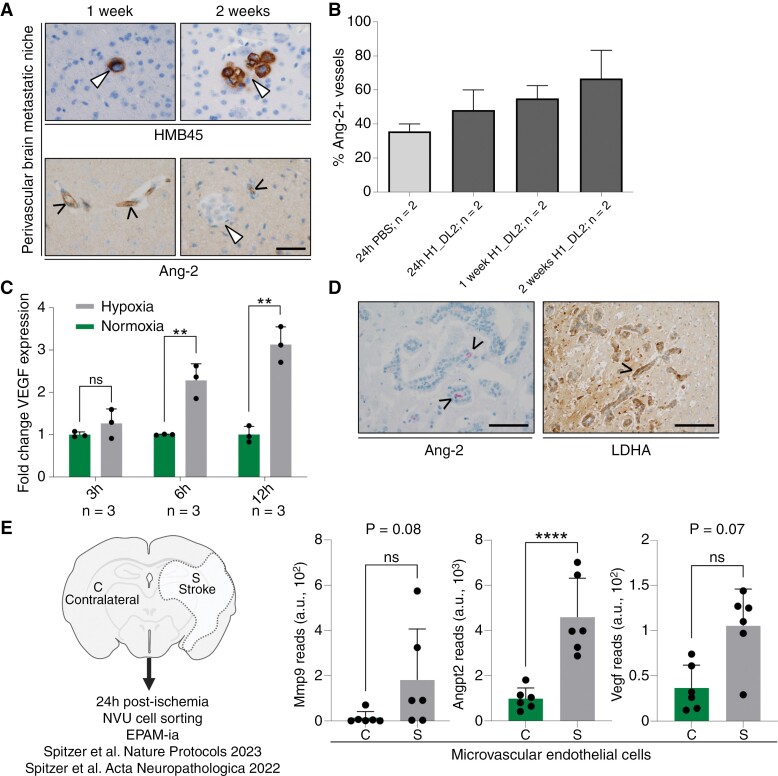
(A) Immunostainings for HMB45 and Ang-2 demonstrating HMB45 expressing melanoma cells, which seed and colonize and form micrometastasis (white arrowhead) close to Ang-2 expressing brain microvessels 1 and 2 weeks after intracardiac injection of tumor cells in mice (scale bar 50 µm). (B) Percentage of Ang-2 expressing brain microvessels of the striatum in animals with intracardiac inoculation of PBS or H1_DL2 melanoma cells. (C) VEGF gene expression in JIMT-1 cancer cells in response to normoxia or hypoxia (** *P* < .01 and ns *P *> .05 by 2-tailed, unpaired *t*-test). (D) Lactate dehydrogenase A (LDHA) protein expression in metastasizing human cancer cells adherent to Ang-2 expressing brain microvessels in the perivascular pre-metastatic niche of a human brain metastasis (left panel scale bar 100 µm, right panel 200 µm). (E) Transcript reads obtained from NVU transcriptome profiling for Mmp9, Angpt2, and Vegf in endothelial cells (c, contralateral; s, stroke; *n* = 6, 3–4 mice per preparartion) [31,32]. ****P < .0001 and ns, not significant determined by DESeq2 with Benjamin-Hochberg correction.

We hypothesized that microvascular cancer cell occlusion and therefore focal alterations of cerebral blood circulation lead to hypoxia and subsequent upregulation of endothelial Ang-2 expression which then supports BM formation. During the observation period between day 1 and day 14, we found Ang-2-expressing vessels close to brain-invading melanoma cells (Figure 2A). In the next step, we investigated Ang-2 expression in striatal microvessels after cancer cell injection. Compared to control animals (intracardiac PBS injection), mice injected with tumor cells exhibited a prominent expression of Ang-2 indicated by an increased number of Ang-2 expressing brain microvessels 24 hours post-injection that continuously increased from days 1 to 14 (Figure 2B). Moreover, exposing metastatic tumor cells to hypoxic conditions in vitro resulted in a steady increase in VEGF mRNA levels over time (Figure 2C). In line with these in vitro experiments, we found that besides Ang-2 expressing endothelial cells, brain metastatic human cancer cells in the pre-metastatic niche strongly expressed lactate dehydrogenase A (LDHA; Figure 2D), a surrogate enzyme for a metabolic deficiency state.^33^

As we observed similarities in the microenvironmental reactions (infarction-like reaction) between early brain colonization and stroke, we analyzed cells isolated from the neurovascular unit from our recently published stroke models for Ang-2, VEGF, and MMP9 expression (Figure 2E).^35,36^ MMP9 has recently been shown by Karreman et al. as being a crucial factor, secreted by cancer cells within a complex extravasation process during brain metastasis formation.^25^ We found all three transcripts upregulated in isolated endothelial cells in the early stroke as compared to the contralateral side (Figure 2E). Ang-2 transcripts were significantly upregulated between stroke and normal contralateral endothelial cells (Figure 2E). Taken together, these findings strongly suggest that microenvironmental changes in early brain metastasis formation might mimic infarction-like microenvironmental reactions. A hypoxic brain microenvironment might promote cancer cell colonization and BM microformation in the pre-metastatic niche by stimulation of Ang-2 and VEGF expression.

### Endothelial Ang-2 GOF Leads to Increased Cancer Cell Extravasation and Increased Brain Metastatic Burden

**Figure 3.**
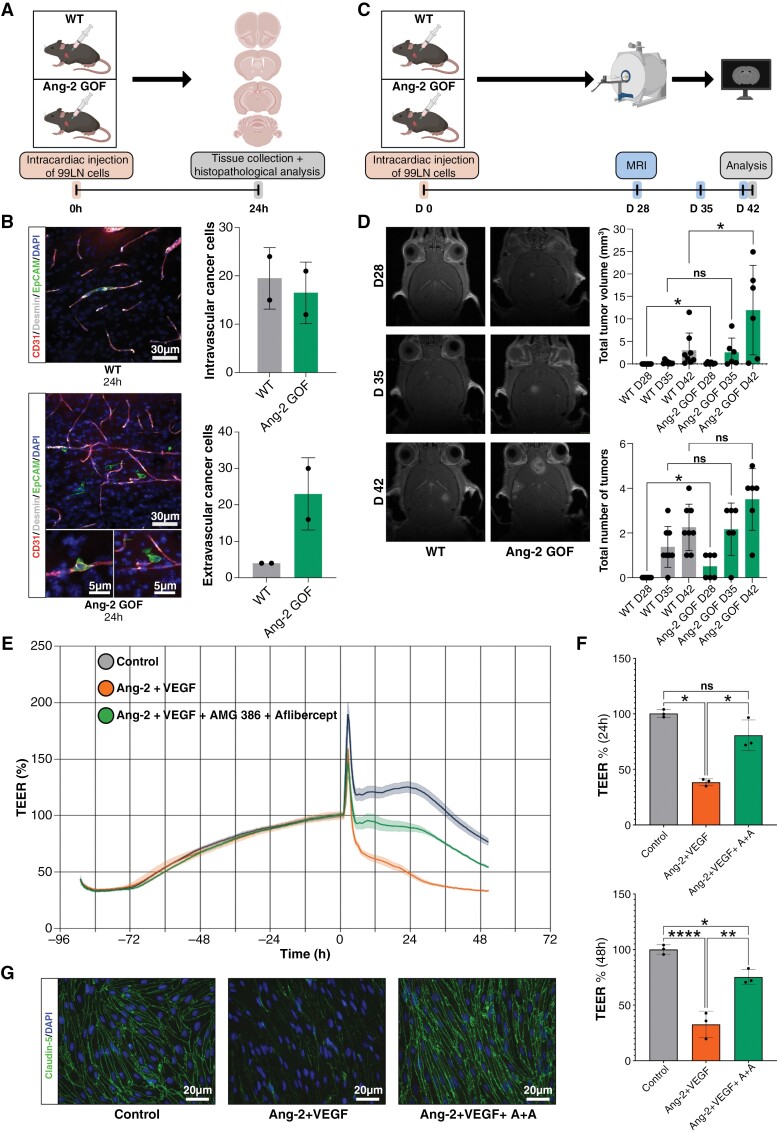
(A) Scheme illustrating intracardiac injection of 99LN cells in wild type (WT) and angiopoietin-2 gain of function (Ang-2 GOF) C57BL/6 mice followed by tissue collection and histopathological analysis 24 hours after intracardiac injection. (B) Representative images of immunofluorescence staining for CD31 and desmin expressing microvessels and epithelial cell adhesion molecule (EpCAM) expressing tumor cells in WT and Ang-2 GOF C57BL/6 mice demonstrating intravascular and extravascular tumor cells (left panel); immunofluorescent analysis of 50 µm vibratome sections of WT and Ang-2 GOF mice (*n* = 2 WT and *n* = 2 Ang-2 GOF) revealed an increased migration and invasion of 99LN cells in Ang-2 GOF mice (right panel). (C) Schematic illustration of brain metastasis evolution experiment, depicting intracardiac injection of 99LN cells in WT and Ang-2 GOF C57BL/6 mice followed by assessment of brain metastatic load by MRI 28, 35, and 42 days after intracardiac injection of tumor cells. (D) MRI imaging revealed increased numbers of tumors and total tumor volumes in Ang-2 GOF mice 28 days and increased total tumor volumes in Ang-2 GOF mice 42 days after intracardiac tumor cell injection; *n* = 8 WT and *n* = 6 Ang-2 GOF, *P < .05 and not significant (ns) *P* > .05 by 2-tailed, unpaired *t*-test. (E) Representative graph for continuous TEER values of the MBMEC monolayer in control conditions and treatment conditions with either Ang-2 and VEGF or Ang-2, VEGF, AMG-386, and Aflibercept. (F) Quantification of 24 and 48 hours TEER values of MBMEC in control conditions and treatment conditions with Ang-2 and VEGF or Ang-2, VEGF, AMG-386, and Aflibercept; *n* = 3 independent experiments, *P < .05, **P < .01, ****P < .0001 and ns *P* > .05 by one way ANOVA (Tukey post hoc test). (G) Representative images of claudin-5 in endothelial monolayer in control and treatment conditions. DAPI was used to reveal cell nuclei.

The upregulation of Ang-2 in a potentially hypoxic pre-metastatic niche suggests a crucial role for Ang-2 in the early brain colonization of cancer cells. Therefore, we investigated 99LN cancer cell colonization in the brain using transgenic Ang-2 GOF mice (Figure 3A). We monitored early brain colonization by termination of animals after 24 hours post intracardiac injection. Using vibratome sections, we were able to quantify intravascular and extravascular cancer cells. Ang-2 GOF mice showed an increased extravasation potential as compared to wild-type animals (Figure 3B). MR imaging of long-term colonization experiments (Figure 3C) revealed an increased tumor volume after 28 and 42 days (Figure 3D) and an increased total number of tumors in Ang-2 GOF mice (Figure 3D). Although this trend was observed during all observational time-points, only the measurement at time-point 28 days p.i. showed statistical significance. These results indicate that extravasation and outgrowth processes of cancer cells in the brain are Ang-2-dependent.

### Ang-2 and VEGF are Strong Modulators of Blood-Brain-Barrier Integrity

As indicated by the proof-of-principle BM experiments Ang-2 GOF revealed that Ang-2 might directly interfere with BBB integrity during brain metastasis formation. We therefore analyzed BBB integrity using TEER measurements of primary mouse brain microvascular endothelial cells (Figure 3E-G). While Ang-2 and VEGF led to a breakdown of BBB capacity, Aflibercept (anti-VEGF) and AMG 386 (anti-Ang-2) were able to rescue this effect (Figure 3E-G). This indicates, that VEGF- and Ang-2 targeted therapies might interfere with cancer cell extravasation and outgrowth processes in the brain.

### Early Dual Inhibition of Hypoxia-Inducible Ang-2 and VEGF Reduces Melanoma and Breast Carcinoma Metastases Burden in the Brain

**Figure 4.**
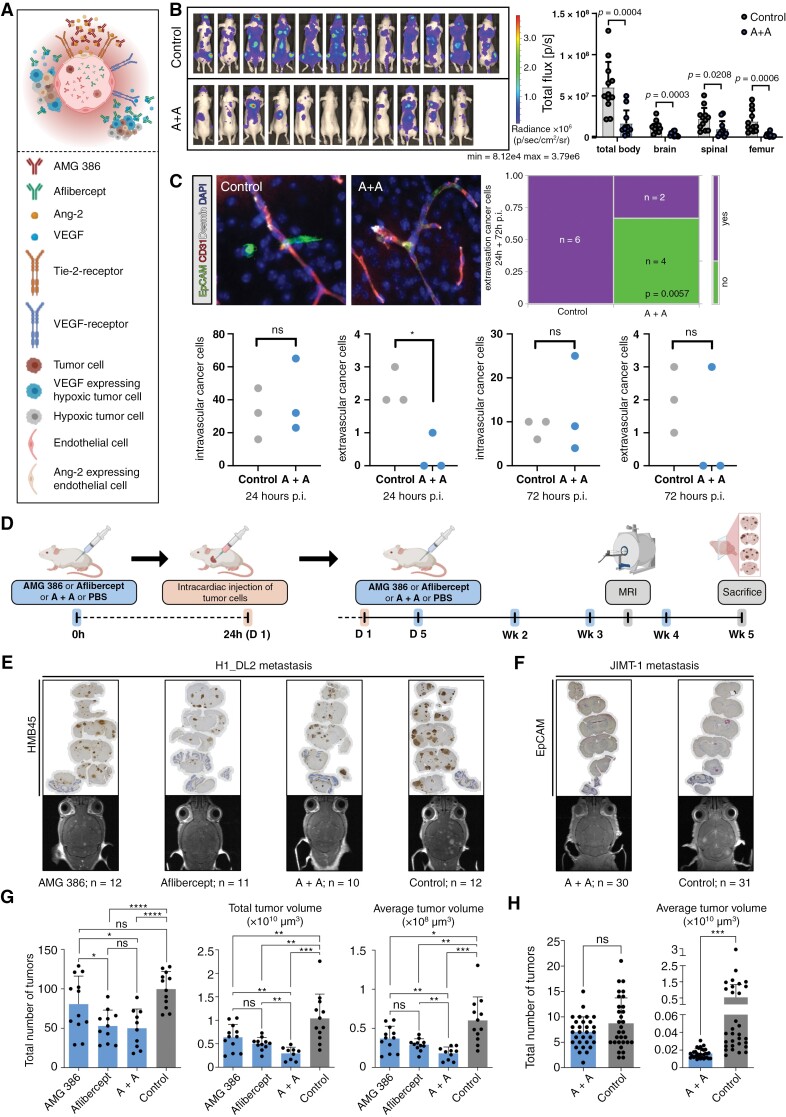
(A) Scheme illustrating microenvironmental Ang-2 and VEGF blocking in brain metastases formation. (B) Bioluminescence imaging 2 weeks after intracardial H1_DL2 injection. Regions of interest “total body,” “brain,” “spinal” and “femur” were defined and measured accordingly. Results of PBS-treated animals (control) and AMG 386 and aflibercept (A + A) treated animals are depicted. Statistical analysis was performed by Student`s *t*-test. (C) Quantification of cancer cell extravasation in vibratome sections of PBS (control) and A + A treated animals are shown after 24 and 72 hours p.i.. The combination of the 24h and 72h p.i. experiments are shown in the upper right panel (purple: extravasation; green: no extravasation, *P*-value of likelihood ratio chi-square test is shown) (D) Experimental design of anti-Ang-2/anti-VEGF treatment in mice with intracardiac injection of H1_DL2 or JIMT-1 cells. (E-H) Assessment of melanoma (E, G) and breast carcinoma brain metastatic load (F, H) by MRI in week 4 after commencement of anti-angiogenic therapies in mice, including the total number of tumors and total/average tumor volumes in animals treated with AMG 386, aflibercept, or AMG 386 plus aflibercept compared to PBS treated controls. Immunostainings of coronal tissue sections for HMB45 (E) and EpCAM (F) visualize cerebral spread of H1_DL2 melanoma and JIMT-1 breast carcinoma metastases 5 weeks after initiation of anti-angiogenic therapies (trial endpoint). * *P *< .05, ** *P *< .01, *** *P *< .001, *P* < .0001 and ns *P *> .05 by 2-tailed, unpaired *t*-test.

Given the early upregulation of VEGF in tumor cells in response to hypoxia in vitro (Figure 2C) and the increased expression of Ang-2 in brain microvessels in the hypoxic pre-metastatic niche (Figure 2A, B, D) demonstrated here, we hypothesized that early therapeutic inhibition of Ang-2 and VEGF-associated microenvironmental changes in hypoxic pre-metastatic niche may substantially affect metastatic growth in the brain (Figure 4A). Therefore, we first analyzed the therapeutic effects of AMG 386 or aflibercept, or AMG 386 plus aflibercept on the human melanoma cell line H1_DL2 and the human breast cancer cell line JIMT-1 in vitro. Importantly, neither the AMG 386 or aflibercept treatment nor the AMG 386/aflibercept combination therapy exerted inhibitory effects on cell proliferation (assessed by crystal violet assay, Supplementary Figure 1C) or MTT cell viability assay (Supplementary Figure 1D). This emphasizes, that this particular treatment strategy selectively targets the tumor microenvironment. Next, we assessed the systemic therapeutic effects of the combination therapy in mice bearing H1_DL2 melanoma cells after intracardiac tumor cell injection. First, using bioluminescent imaging, we observed a significant reduction of H1_DL2 tumor load in the brain, spinal, and femur, suggesting a systemic supportive treatment effect in the animals during combination therapy (Figure 4B). We next focused on brain metastasis. We monitored early cancer cell extravasation using brain vibratome sections 24 and 72 hours post intracardiac injection of JIMT-1 cells. Mice were pretreated with aflibercept and AMG 386 before cancer cell injection. Only time-point 24 hours post intracardiac injection showed significantly reduced extravasated cancer cells in the brain in A + A treated mice (Figure 4C). Interestingly, when combining both experiments (24 + 72 hours) all investigated specimens showed extravascular cancer cells in the control animals (PBS treated) while in the A + A condition, 4 animals did not show signs of cancer cell extravasation (Figure 4C). These results underline, that cancer cell extravasation depends on Ang-2 and VEGF and therefore preventive strategies targeting both molecules might improve therapy efficacy. Therefore, we further validated brain metastasis colonization by MRI and immunohistochemistry. Animals were pretreated with AMG 386 or aflibercept, AMG 386 plus aflibercept or PBS (controls) followed by intracardiac inoculation with melanoma cells (Figure 4D, left part of the panel). Anti-angiogenic treatment was continued 5 days after tumor cell injection followed by injections of the anti-angiogenic drugs twice a week (Figure 4D, right part of the panel). Metastatic burden and size of metastatic lesions in the brain were measured by MRI in week 4 after tumor cell injection. Immunostainings of brain tissue sections were performed 5 weeks after the commencement of the therapies (Figure 4E). Evaluation of the metastatic brain with MRI revealed a significant decrease in tumor numbers and tumor volumes in animals treated with AMG 386 or aflibercept alone, or AMG 386 plus aflibercept (A + A) compared to controls receiving PBS injections (Figure 4G). Among the tested treatment regimes, the AMG 386/aflibercept combination therapy unfolded the strongest inhibitory effects on brain melanoma metastases load, except for reduction of the total number of metastatic brain tumors that was comparable with that of the aflibercept monotherapy (Figure 4G). Given the potent inhibitory effect of the combined anti-angiogenic therapy on melanoma BM development in vivo, we administered AMG 386 plus aflibercept (A + A) to mice that were intracardially injected with JIMT-1 breast cancer cells, according to the treatment strategy demonstrated in Figure 4D. Anti-angiogenic combination therapy in the breast cancer model, which usually shows rather large but few BM, resulted in a slightly decreased number of metastatic brain tumors and decreased tumor volumes compared to controls (Figure 4F, H). However, a significant difference was only observed for tumor volumes (Figure 4H).

Taken together, these findings propose that the combined anti-Ang-2/anti-VEGF therapy may ameliorate Ang-2 and VEGF-mediated microenvironmental changes in hypoxic perivascular niches that are essential for successful metastatic tumor outgrowth in the brain.

### Ang-2 Expression in Established Human BM and Association With Clinical and Biological Parameters

**Figure 5.**
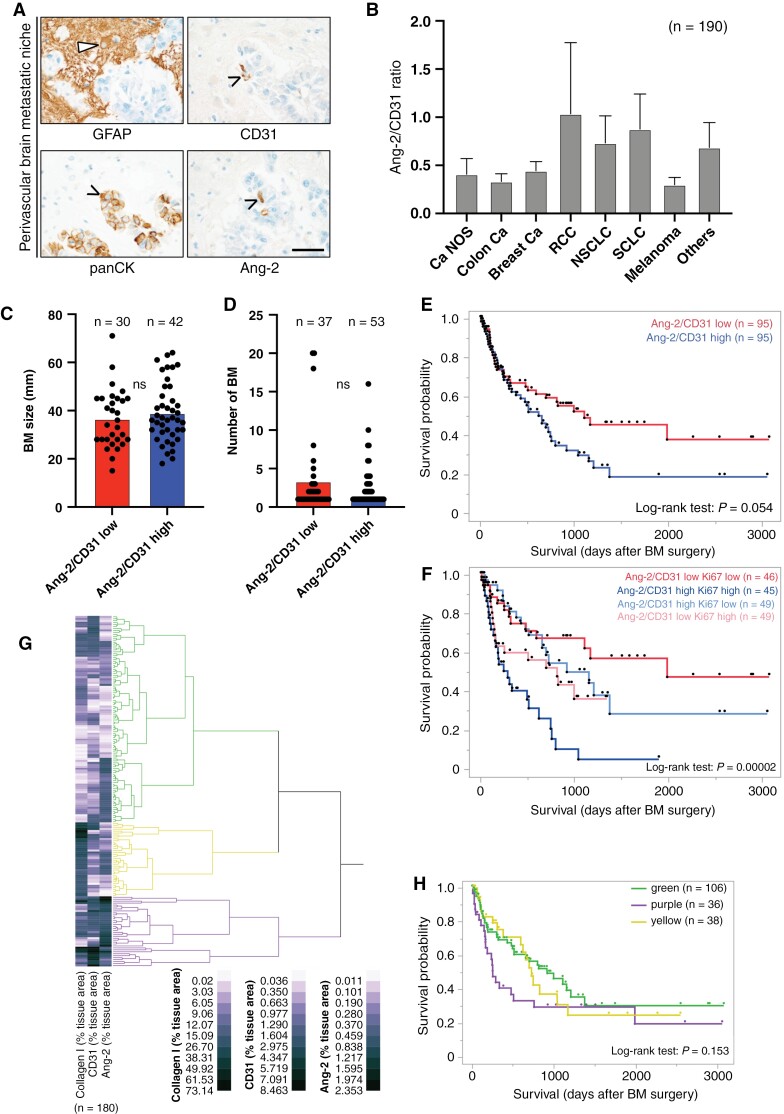
(A) Immunostainings demonstrate Ang-2 expression in CD31-positive brain microvessels exploited by panCK-positive metastasizing cancer cells in the human brain pre-metastatic niche (scale bar 50 µm). (B) Ratio of Ang-2/CD31 expression in tumor vessels in human brain metastases (BM) of different types of metastatic cancers (*n* = 191 patients Ang-2 data, one patient missing CD31 data). (C) Association of Ang-2/CD31 ratio and BM size. (D) Association of Ang-2/CD31 ratio and number of BM. (E) Kaplan–Meier survival curves of patients with low or high ratios of Ang-2/CD31 expression (median split) in metastatic brain lesions. Results of Log-rank test are shown. (F) Kaplan–Meier survival curves of combined Ki67 dichotomization (median split) and Ang-2/CD31 ratio dichotomization (median split) showing four groups illustrating low/low, high/high, high/low, and low/high expressors (total *n* = 189 due to single patients with missing data for CD31 and Ki67). Hierarchical cluster analyses of tumor area, positively labeled with antibodies against collagen I, CD31, and Ang-2. Kaplan–Meier survival curve of patients belonging to different stromal vascular clusters (H). Survival from date of BM surgery until last contact (H) are depicted. Throughout the figure, the numbers of the samples tested are indicated in parentheses. These vary due to partly missing clinical or experimental data.

To further evaluate the clinical relevance of Ang-2 in the microvasculature, we investigated TMAs of BM from 191 patients diagnosed with brain metastasis and used them to assess Ang-2 protein expression in blood vessels (Figure 5A). Interestingly, we identified a high Ang-2 protein expression not only in tumor vessels but also in preexisting brain microvessels that were strongly co-opted by metastatic cancer cells (Figure 5A). Importantly, tumor cells did not show Ang-2 expression in the analyzed samples. Subsequent quantification of the ratio of Ang-2 expression relative to the expression of CD31 revealed a markedly increased Ang-2 expression in vessels of all screened types of BM. We observed the highest Ang-2/CD31 ratio in vessels of NSCLC and SCLC (Figure 5B). RCCs are typically highly vascularized and therefore showed high, but also highly variable Ang-2 expression. Although we observed a slight trend for increased size of Ang-2/CD31 high brain metastasis, we did not observe statistical significance (Figure 5C). The number of BM was not associated with Ang-2/CD31 ratio of resected tumors (Figure 5D).

Importantly, the increased Ang-2/CD31 ratio detected in patients suffering from BM negatively correlated with patient survival after tumor resection, suggesting that increased expression of Ang-2 overall and in the pre-metastatic niche substantially contributes to disease progression (Figure 5E). When we added the tumor’s proliferative activity, as measured by the Ki67 proliferation rate, to the survival analyses, we found that the combination of Ang-2/CD31 ratio and Ki67 was strongly associated with survival following BM surgery (Figure 5F). Furthermore, when investigating the stromal compartment of established brain metastasis using antibodies against Collagen I, Ang-2, and CD31, we observed three major clusters of brain metastasis (Figure 5G) with variable expression profiles. Most interestingly, the purple cluster (high Ang-2 and high CD31 levels with moderate collagen I expression) was negatively associated with overall survival after brain surgery (Figure 5H). This suggests, that also in established brain metastasis Ang-2, Ki67, and a certain composition of the stroma-vascular compartment are associated with progressive disease. Extended survival analyses and cohort statistics are shown in Supplementary Table 1 and Figure 2.

## Discussion

Metastasis is one of the major challenges in oncology. Especially, in brain metastasis, the understanding of the homing processes of cancer cells and colonization of the brain metastatic niche is of paramount importance, as most drugs do not cross the BBB and do not reach the brain parenchyma in sufficient concentrations to exert clinically meaningful anti-tumor effects.

**Figure 6.**
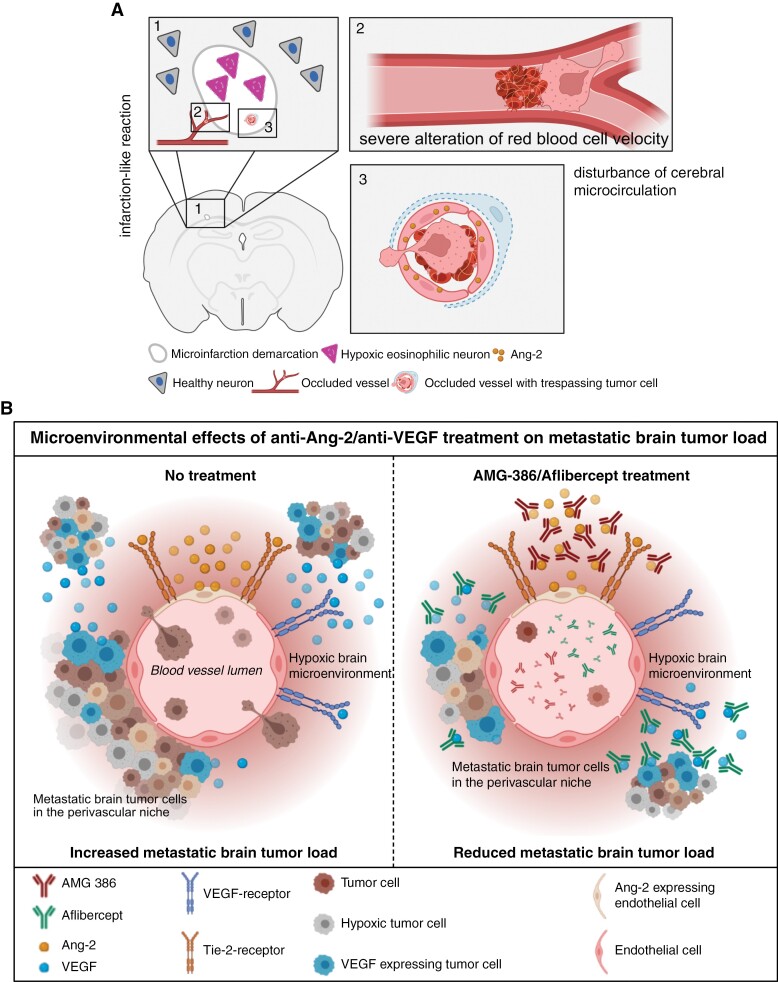
Schematic summary illustrating the microenvironmental effects of (A) cancer cells occluding brain blood vessels and (B) early anti-Ang-2/anti-VEGF treatment on brain metastatic (BM) tumor load.

In our study, we identified focal hypoxic and ischemic changes in the brain pre-metastatic niche as a result of cancer cell vessel occlusions, which lead to an upregulation of vascular remodeling factors such as Ang-2, thereby facilitating the trespassing and colonization of cancer cells in the brain (Figure 6). The observation of cancer cells occluding vessels in our experimental setup was accompanied by a focal alteration of blood velocity which somehow mimicked the process of early stroke. This has been confirmed by comparing Ang-2, VEGF, and MMP-9 expression profiles in isolated endothelial cells from experimental stroke.^35^ Interestingly, in mice intracardially injected with cancer cells, we detected microinfarctions which showed at least a partial overlap with areas of later metastatic foci. In these regions, we also detected Ang-2 overexpressing brain microvessels which were in close contact with early tumor cell colonies. Early expression of Ang-2 in stroke has been described by Beck et al.^37^ Furthermore, Ang-2 GOF experiments in murine vasculature led to impairment of the BBB, which was rescued by targeting Tie2 in a middle cerebral artery occlusion model (MCAO).^29^ So far, it is unclear, whether a complete infarction-like lesion is necessary for the process of cancer cell extravasation into the brain. We rather assume, that also transient infarction-like microenvironmental processes are sufficient to induce destabilization of the BBB and preparation of a pre-metastatic niche.

Ang-2 is an important angiogenic regulator that is critically involved in destabilizing the microvasculature and promoting angiogenesis by competing with angiopoietin-1 for Tie-2 receptor binding.^38,39^ As such it has been identified as a key regulator of cancer cell propagation and tumor growth in several types of cancer, including breast and colorectal cancer, renal cell carcinoma, and melanoma.^40–43^ Furthermore, Ang-2 is critically involved in the development and outgrowth of brain cancer, including primary brain tumors such as glioblastoma,^20,21^ and BM. Particularly in breast carcinoma, Ang-2 contributes to colonization of circulating cancer cells in the brain during the early stages of the metastatic cascade in a mouse model of breast cancer metastasis.^5^ These findings reflect our observations in patients diagnosed with BM, showing increased Ang-2 expression in brain microvascular endothelial cells already at the early stages of brain colonization. These results suggest that Ang-2 upregulation in brain microvascular endothelial cells may be crucially involved in the early development of BM in several types of cancer. Moreover, we found endothelial Ang-2 expression in microvessels of solid brain metastatic tumors. These findings suggest that Ang-2 on the one hand might be a potent driver of tumor cell transmigration through the vascular wall and colonization in the perivascular pre-metastatic niche in the brain and on the other hand ensures stable oxygen and nutrient supply to growing macrometastases. Our data suggests that these results might be regardless of the type of cancer that metastasizes to the brain. The combination of high Ang-2 expression in endothelial cells of macrometastases and the high proliferative capacity of the tumors was associated with decreased survival probability. In these late stages of metastasis outgrowth, we furthermore detected, that the composition of the stroma-vascular compartment is crucial for brain metastasis patient prognosis. The Ang-2 GOF experiments revealed increased brain metastasis sizes and higher extravascular cancer cell counts in the brain parenchyma of endothelial Ang-2 GOF mice. These results implicate a dual function of Ang-2 in the early metastatic stage as well as in the formation of macrometastases.

Our assumption that Ang-2 overexpression in human and murine brain capillaries crucially promotes cancer cell colonization in the brain is further supported by similar observations made in breast cancer BM. In these in vivo mouse models, increased Ang-2 expression in brain capillary endothelial cells was directly associated with BBB disruption, further facilitating extravasation and colonization of circulating breast cancer cells in the pre-metastatic niche.^5^ Moreover, as expected, we observed a substantial upregulation of VEGF in cancer cells in vitro in response to hypoxia. Our results suggest that a hypoxic environment may further contribute to successful cancer cell propagation in perivascular pre-metastatic niches by increasing Ang-2 expression in brain microvessels and VEGF expression in brain-invading tumor cells.^14–17,44^ It is of note that hypoxic conditions may be well tolerated by brain-invading cancer cells, particularly as we detected an increased expression of lactate dehydrogenase A (LDHA) also in early metastatic cancer cells. Ang-2 expressing microvessels, which support nutrition supply in combination with a metabolic switch in cancer cells towards a glycolytic phenotype in an ischemic pre-metastatic niche, might support cancer cell survival and the establishment of solid macrometastases. Interestingly, the knockdown of LDHA was not capable of reducing brain metastasis colonization in a melanoma BM model.^33^ A recent study showed that early platelet activation and clot formation at metastatic sites were crucial for successful brain metastatic colonization.^45^ This supports our hypothesis of early infarction-like reactions as the driving force of tumor cells seeding to the brain.

Upregulation of Ang-2 and VEGF has been demonstrated to significantly contribute to tumor growth^16,46^ and metastases formation.^5,13^ In preclinical studies dual inhibition led to improved outcomes in different tumor entities.^16,17,47–50^ However, the effect of early dual inhibition of Ang-2 and VEGF on BM development in mice suffering from melanoma and breast cancer using the Ang-2 inhibitor AMG 386 and the VEGF-trap aflibercept has not been investigated yet. Importantly, toxic effects on both types of tumor cells were not recorded at any time point investigated in in vitro experiments by either treatment (AMG 386 or aflibercept) alone or in combining both. We were able to show, that Ang-2 and VEGF disrupt BBB integrity and that AMG 386 and aflibercept are able to rescue this effect. Therapeutic inhibition of Ang-2 and VEGF in mice intracardially injected with either melanoma or breast cancer cells, was associated with a substantial reduction of the metastatic brain tumor load by either AMG 386 or aflibercept administration alone or by dual anti-Ang-2/anti-VEGF therapy. The most beneficial effects were achieved by early dual anti-Ang-2/anti-VEGF therapy, further underpinning that microenvironmental changes towards a supporting microenvironment—a pre-metastatic niche, may be substantially mediated by Ang-2 and VEGF.

In conclusion, we here provide evidence for a novel mechanism of how cancer cells shape a pre-metastatic niche by blood vessel occlusion and subsequent focal infarction-like microenvironmental reaction, which in turn leads to an upregulation of vascular remodeling factors such as Ang-2 or VEGF. In addition, Ang-2 might be involved in a delayed resolution of cancer cell-blood clotting, as described for venous thrombosis.^51^ Furthermore, our findings support previous preclinical studies, which demonstrated that both Ang-2 and VEGF are crucially involved in BM formation.^5,13^ An important clinical question the present work does not address, is the role of radiotherapy in the context of cancer cell extravasation and in the context of the proposed mechanism. Radiotherapy not only directly targets proliferating cancer cells but also influences cerebral blood microcirculation.^52^ Further studies are needed to address this question. Although we have discovered interesting and promising results regarding Ang-2 as a potential prognostic marker in established macrometastases in BM patients, these data should be interpreted with caution. Firstly, our study involves a very selected and heterogeneous cohort of patients (all patients underwent resection), and secondly, the case numbers of the individual tumor entities examined were partly very low, so that conclusive interpretations and clear applications to the clinical situation are not definitively possible at the moment.

Early inhibition of reprogramming effects of Ang-2 and VEGF on the brain perivascular pre-metastatic niche by combined AMG 386/aflibercept therapy is beneficial in preclinical xenograft models of breast carcinoma and melanoma BM, demonstrating a substantial reduction of the brain metastatic tumor load. Our findings suggest that combined inhibition of Ang-2 and VEGF can to a certain extent prevent brain metastasis by keeping metastatic tumor cells dormant and in a clinically silent state. Future research is warranted on the pre-metastatic niche as a sanctuary side of metabolically derailed tumor cells, and of the identification of patients at high risk to develop BM which may benefit from prevention therapies.

## Supplementary material

Supplementary material is available online at *Neuro-Oncology* (https://academic.oup.com/neuro-oncology).

noae094_suppl_Supplementary_Figure_S1

noae094_suppl_Supplementary_Figure_S2

noae094_suppl_Supplementary_Material

noae094_suppl_Supplementary_Table_S1

noae094_suppl_Supplementary_Video

## Data Availability

The data that support the findings of this study are available from the corresponding author upon reasonable request.
