# Supplementary figures and images for: Disturbance in cerebral blood microcirculation and hypoxic-ischemic microenvironment are associated with the development of brain metastasis
Source: Neuro Oncol. 2024 Jun 4;26(11):2084–99. doi: 10.1093/neuonc/noae094 (PMC11534324; doi:10.1093/neuonc/noae094)

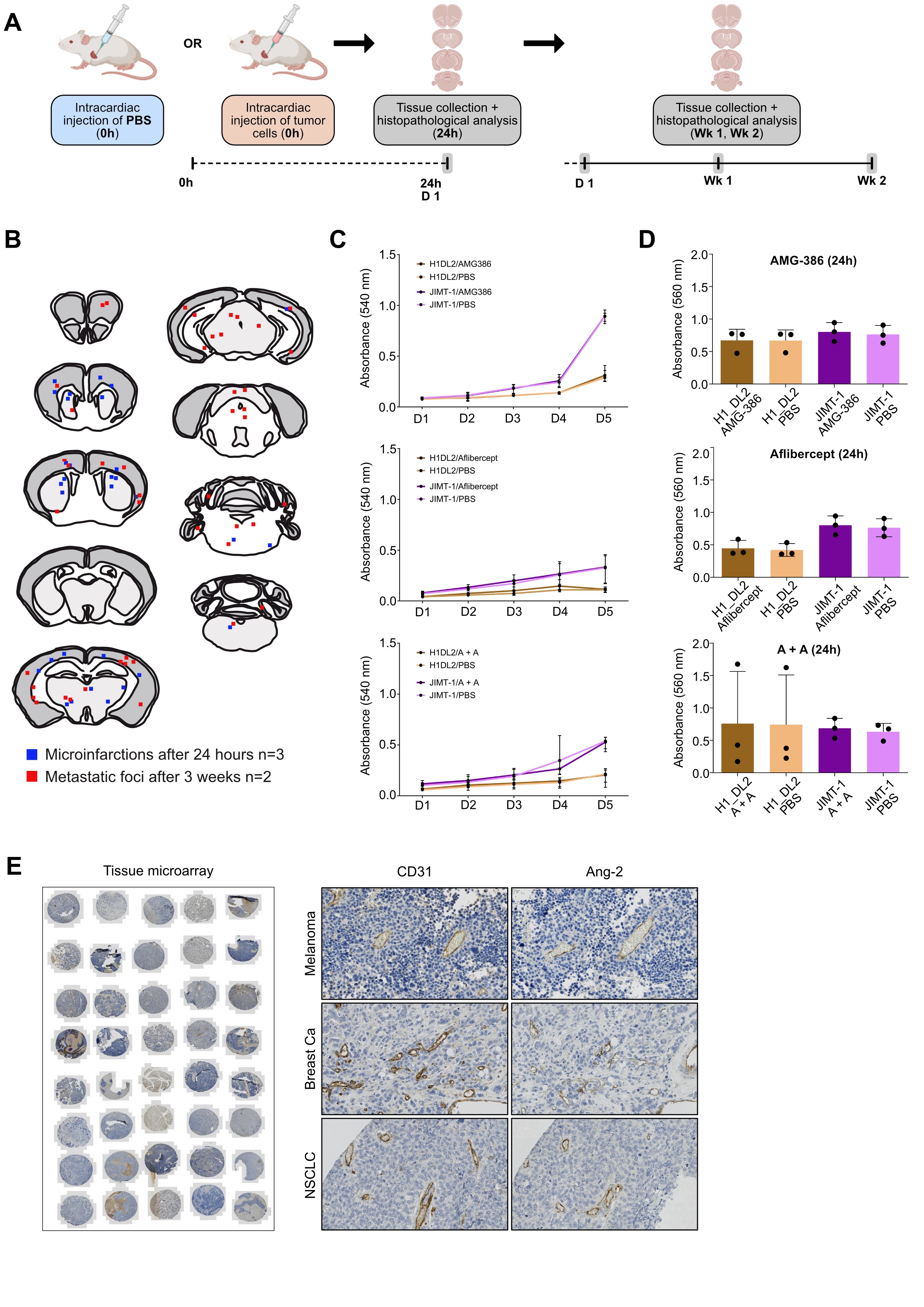

Supplement: noae094_suppl_Supplementary_Figure_S1 [file noae094_suppl_supplementary_figure_s1.jpeg]

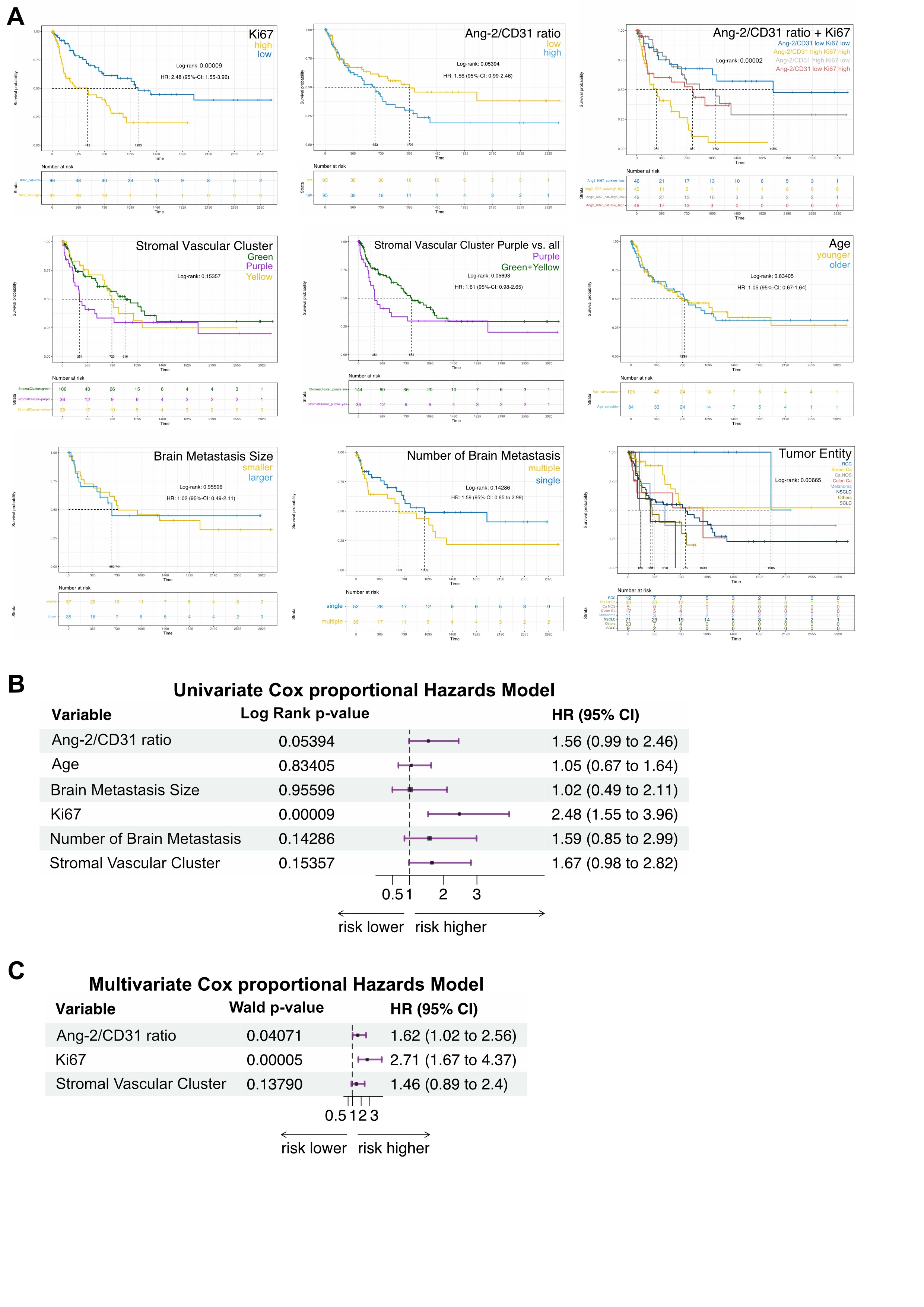

Supplement: noae094_suppl_Supplementary_Figure_S2 [file noae094_suppl_supplementary_figure_s2.jpeg]
