# Supplementary material for: Disturbance in cerebral blood microcirculation and hypoxic-ischemic microenvironment are associated with the development of brain metastasis
Source: Neuro Oncol. 2024 Jun 4;26(11):2084–99. doi: 10.1093/neuonc/noae094 (PMC11534324; doi:10.1093/neuonc/noae094)
Supplement: noae094_suppl_Supplementary_Material [file noae094_suppl_supplementary_material.docx]

**Supplementary methods:**

**Animal care and handling**

The number of animals was kept to a minimum based on statistically appropriate sample size. All experiments using animals were strictly conducted in accordance with the German Protection of Animals Act and in compliance with the recommendations in the Guide for Care and Use of Laboratory Animals of the National Institutes of Health and were approved by the local governmental authorities (Regierungspraesidium Darmstadt, Germany; approval number FK/ 1085). For MR scans, mice were continuously anaesthetized with isoflurane (2%) and stabilized in the prone position with a tooth holder. Body temperature was kept at 37°C with a built-in animal waterbed (Bruker, Ettlingen, Germany) and respiration rate was monitored and kept constant at 80-100 breaths per minute while decreasing or increasing the gas anaesthesia. All animals suffering from metastasis were sacrificed when they developed dramatic weight loss (>20% of body weight) or started to show neurological symptoms.

Multiphoton Laser Scanning Microscopy (MPLSM) imaging: all animal procedures were performed in accordance with the institutional laboratory animal research guidelines after approval of the Regierungspräsidium Karlsruhe, Germany (governmental authority; 35-9185.81/G-220/16 and 35-9185.81/G-273/19). All efforts were made to minimize animal suffering and to reduce the number of animals used according to the 3R’s principles. Mice were routinely checked for clinical endpoint criteria.

**Cell lines and culture**

The brain-homing melanoma cell line H1_DL2 was derived from a human brain metastasis (provided by Frits Thorsen, Bergen, Norway). JIMT-1 brain homing breast carcinoma cell lines were a gift from Patricia Steeg and Frank Winkler. The cell lines were maintained in DMEM GlutaMax (Invitrogen) with 10% fetal bovine serum (FBS Superior, Biochrome) and 1% penicillin/streptomycin (P/S, Sigma-Aldrich) in a humidified atmosphere (+37°C, 5% CO2). Syngeneic experiments were performed using the murine breast cancer brain homing cell line 99LN.

**Blood velocity measurement and 2-photon microscopy**

For the induction of anaesthesia, mice were exposed to 3-5% isoflurane in 100% O2, which was lowered to 0.5-2% for anaesthesia maintenance. During imaging, body temperature of the mice was monitored and kept at 37°C using a temperature sensor and a heating plate. Anaesthesia was regularly evaluated during image acquisition by checking postures and breathing rates. FITC-dextran (fluorescein isothiocyanate-Dextran, 500.000 g/mol) was dissolved in 0.9% NaCl-solution at 10 mg/ml. 100 µl of FITC solution was injected into the lateral tail vein for blood vessel visualization. Mice were imaged using a Zeiss LSM 980 equipped with a tunable femtosecond laser (Discovery NX; Coherent) and a 20×, 1.0 NA, apochromatic, 1.7 mm working distance, water immersion objective (Zeiss). tdTomato-expressing tumor cells and FITC dextran were imaged using a 950 nm excitation wavelength. For every object, > 10.000 linescans (~5s) were acquired parallel to the blood flow proximal and distal of the object, and in a neighboring, independent microvessel of similar size and shape. Erythrocytes passing through the vessels cast a shadow in the linescan and thus allow for the calculation of corpuscular blood flow velocity.

**Cell viability and toxicity assays**

For CV assays, human brain metastatic tumor cell lines H1_DL2 and JIMT-1 were seeded in 96-well plates (1,000 cells/well) and incubated in a humidified atmosphere (+37°C, 5% CO2) for 24 hours. After a 24-hour incubation period, medium was aspirated and fresh medium supplemented with the therapeutic agents was added to each well. In the treatment groups, cells were either subjected to AMG 386 (500 ng/mL) or aflibercept (1 mg/mL) alone, or to a combination treatment with AMG 386 and aflibercept (A+A, 500 ng/mL + 1 mg/mL), whereas cells in the control group were subjected to PBS. Cells in each group were then incubated in a humidified atmosphere (+37°C, 5% CO2) for 24 and 48 hours. After 24 h and 48 h of incubation, respectively, the medium was removed and a total of 50 µL of 0.5% CV staining solution in 25% methanol was added to each well after washing and drying. Sorenson’s buffer (50% ethanol and 0.1 M sodium citrate) was added at a concentration of 50 µM per well, and the plates were agitated on a bench rocker for 30 min at room temperature. Optical density of each well was measured spectrophotometrically at 540 nm using a microplate reader (TECAN reader infinite M200 pro). For MTT reduction assay, tumor cell lines H1_DL2 and JIMT-1 were seeded in full media in 96-well plates (10,000 cells/well) applying the same culture and treatment protocols as stated above for the CV assay. After incubation of cells in a humidified atmosphere (+37°C, 5% CO2) for 24 and 48 hours, the medium was removed and a total of 25 µL of MTT solution containing 5.0 mg MTT/mL was added to each well. Plates were then incubated in a humidified atmosphere (+37°C, 5% CO2) for 2 hours. Next, 100 µL of lysis buffer (100 mL of DMF, 100 mL of aqua dest, 40 g of SDS, 2.5 mL of 80% acetic acid and 2.5 mL of 1 M HCL) was added to each well to extract MTT-formazan cristals from the cells. After overnight incubation of cells in a humidified atmosphere (+37°C, 5% CO2), the absorbance of the formazan product was measured at 560 nm using a microplate reader (TECAN reader infinite M200 pro).

**Real-Time quantitative PCR (RT-qPCR), hypoxia experiments**

The concentration of total RNA was determined photometrically with the NanoDrop™ 2000 spectral photometer (Thermo Scientific, Dreieich, Germany). Reverse transcription of 1µg of RNA into complementary DNA (cDNA) was performed according to the manufacturer’s protocol of the RevertAidTM H Minus First Strand cDNA synthesis Kit (Thermo Scientific, Dreieich, Germany) using random hexamer primers. The RT-qPCR was performed by combining SYBR green master mix (Thermo Fisher Scientific, Waltham, MA, USA) and the forward and reverse primers of Vegf (Sequence 5’-3’ sense: AGCCTTGCCTTGCTGCTCTA, Sequence 5’-3’ antisense: GTGCTGGCCTTGGTGAGG) in a final volume of 20 µl and measuring Vegf mRNA levels on a MyiQ Single Color Real-Time PCR Detection System (BIO-RAD, Hercules, CA, USA). Ribosomal protein lateral stalk subunit P0 (Rplp0; Sequence 5’-3’ sense: TCCGACTCTTCCTTGGCTTCA, Sequence 5’-3’ antisense: GAGTCCTGGCCTTGTCTGTGG), a house-keeping gene, was used as internal control.

**Slice culture and quantification of extravasation**

After intracardiac inoculation of cancer cells, animals were terminated at the respective time points. Brains were sectioned into 50µm thick slices using a vibratome. Immunofluorescence staining for CD31 and desmin expressing microvessels were performed. Epithelial cell adhesion molecule (EpCAM) was used for the detection of cancer cells, 4′,6-Diamidino-2-phenylindole (DAPI) was used for the detection of cell nuclei. Intravascular and extravascular cells were quantified according to their localization in the tissue.

**Quantification of intravascular cancer cell thrombi**

We measured n=30 tumor cells (both lines H1 and JIMT-1) from in total n=3 mice. For JIMT-1, we used a correlative dataset by Karreman et al. (PMID: 36652557) in which electron microscopy on mice brains was performed after the animals were imaged first with intravital microscopy. For quantification, we analyzed cancer cell thrombi, that were proven single cells by electron microscopy. The cancer cell volumes were measured using the images of intravital microscopy. We measured single cell volumes of JIMT-1 (n=5 cells) in comparison to all investigated JIMT-1 (n=30 cells) and H1 cells (n=30 cells).

**MR Imaging parameters**

For JIMT-1 cells, we used the following parameters: Mice were injected intraperitoneally (i.p.) with 150 µl Gadobutrol (Gadovist, 1 mmol/ml, Bayer) before the MR imaging T1: TR 1500ms; TE 6.5ms; Matrix size 256 × 256; Slice thickness 0.5 mm. T2: TR 2500 ms; TE 33 ms; Matrix size 256 × 256; Slice thickness 0.5 mm; FOV: 2.00 cm. For H1_DL2 we used the following parameters: A T2 weighted scan was performed in coronal orientation (TR: 3200 ms, TE: 38 ms, FOV: 2.00 cm, matrix size: 256 × 256, slice thickness: 0.5 mm, number of slices: 12 and NEX: 4) followed by T1 scans using the same geometrical parameters (TR: 1000 ms, TE: 9 ms, and NEX: 4) pre- and post- administration of 0.5 mmol/kg Omniscan (GE Healthcare).

**Immunohistochemistry and primary antibodies**

Brain tissue samples of 191 patients included in this study for histopathological analysis comprised of tissues from metastatic lesions of carcinoma not otherwise specified (NOS, n=5), colon carcinoma (n=17), breast carcinoma (n=43), renal cell carcinoma (RCC, n=12), non-small cell lung cancer (NSCLC, n=71), small cell lung cancer (SCLC, n=9), melanoma (n=11) and rare entities, such as single cases of ovarian carcinomas, uterine carcinomas or upper gastrointestinal tumors, were referred to as “others” (n=23). Immunohistochemical staining was performed based on standardized protocols of the Leica BOND III automated staining device (Leica, Leica Mikrosysteme Vertrieb GmbH, Wetzlar, Germany). The automated deparaffinization/rehydration, citrate buffer-based antigen retrieval, and blocking of unspecific protein binding and endogenous peroxidase were followed by incubation with primary antibodies. We used monoclonal mouse anti-CD31 (clone JC70A, 1:200, Dako), monoclonal rabbit anti-Ang-2 (#PA5‐27297, 1:200), monoclonal mouse anti-panCK (clone MNF116, 1:1000, Dako), polyclonal rabbit anti-GFAP (1:14000, Dako), polyclonal rabbit anti- EpCAM (1:2000, Abcam), monoclonal mouse anti-HMB-45 (1:100, Cell Marque), monoclonal rabbit anti-LDHA (clone C4B5, 1:100, Cell Signaling), monoclonal mouse anti-Desmin (clone D33, 1:300, DAKO), monoclonal rabbit anti-COL1A1 (Clone E8I9Z, 1:500, Cell Signaling Technology, USA), mouse anti-Ki67 (Clone MIB-1, 1:200, DAKO). Primary antibody binding to tissue sections was visualized either using the BOND Polymer Refine Detection System (substrate chromogen 3,3’-Diaminobenzidine tetrahydrochloride hydrate (DAB), brown precipitate) or for immunofluorescence (IF) labeled secondary antibodies. Counterstaining for IF was performed with 4,6-Diamidine-2´-phenylindole dihydrochloride (DAPI; Invitrogen). IHC stainings were analysed using a light microscope (BX41, Olympus, Hamburg, Germany). Additionally, immunostained human and mouse brain tissue sections were scanned with an automated slide scanner (Zeiss Axio Scan.Z1) and analysed using the HALO-software tool (2.3, indica lab, Albuquerque, NM).

**Transendothelial electrical resistance (TEER)**

Murine brain microvascular endothelial cells (MBMECS) were isolated, cultured and seeded at 100.000/sq.cm in fibronectin coated 24 well PET inserts with 1 micron pore size in medium comprising MCDB-131 complete medium. One hour after plating, the transwell inserts were transferred to a CellZscope device and measurements were continued for upto a week. Once the cells reached confluence indicated by plateau in the TEER graphs, treatments were performed in MCDB-131 basal medium comprising just Pen-Strep (1X) and L-glutamine (2mM). VEGF was used at 20ng/ml, Ang-2 at 500ng/ml, Amg-386 at 30ug/ml and Aflibercept at 25ug/ml). For quantifications, 3 independent preps were performed using 8-10 adult C57BL/6 mice each time. The pretreatment values were set to 100% within the CellZscope software and data exported to spreadsheet for normalization to controls from the 3 sets, that were then analyzed for statistical significance by one way anova (Tukey posthoc test) in PRISM (version 9).
