# Supplementary material for: Disturbance in cerebral blood microcirculation and hypoxic-ischemic microenvironment are associated with the development of brain metastasis
Source: Neuro Oncol. 2024 Jun 4;26(11):2084–99. doi: 10.1093/neuonc/noae094 (PMC11534324; doi:10.1093/neuonc/noae094)
Supplement: noae094_suppl_Supplementary_Table_S1 [file noae094_suppl_supplementary_table_s1.docx]

| Entity distribution (n=191) | Ca NOS n=5  Colon Ca n=17  Breast Ca n=43  RCC n=12  NSCLC n=71  SCLC n=9  Melanoma n=11  Others n=23 |
| --- | --- |
| Sex distribution (f/m) | f: n=107 / m: n=84 |
| Patient age at the time of brain surgery (years) | 13-80 years median 62 years |
| Number of brain metastasis | 1-20 median 1 brain metastasis |
| Size of brain metastasis (diameter mm) | 15-71 mm median 36 mm |
| Follow-up brain surgery until last contact | 0-3071 days median 237 days |
| Follow-up primary cancer diagnosis until last contact | 0-15479 days median 882 days |
